# Effects of *Momordica charantia* (Bitter Melon) on Ischemic Diabetic Myocardium

**DOI:** 10.3390/molecules22030488

**Published:** 2017-03-20

**Authors:** Attila Czompa, Alexandra Gyongyosi, Kitti Szoke, Istvan Bak, Evelin Csepanyi, David D. Haines, Arpad Tosaki, Istvan Lekli

**Affiliations:** Faculty of Pharmacy, Department of Pharmacology, University of Debrecen, Debrecen 4032, Hungary; czompa.attila@pharm.unideb.hu (A.C.); gyongyosi.alexandra@pharm.unideb.hu (A.G.); szoke.kitti@pharm.unideb.hu (K.S.); bak.istvan@pharm.unideb.hu (I.B.); csepanyi.evelin@pharm.unideb.hu (E.C.); donald.david.haines@pharm.unideb.hu (D.D.H.); tosaki.arpad@pharm.unideb.hu (A.T.)

**Keywords:** ischemia, diabetes, bitter melon

## Abstract

*Objective*: A rat model is here used to test a hypothesis that *Momordica charantia* (Bitter melon (BM)) extract favorably alters processes in cardiovascular tissue and is systemically relevant to the pathophysiology of type 2 diabetes (T2DM) and related cardiovascular disease. *Methods*: Male Lean and Zucker Obese (ZO) rats were gavage-treated for six weeks with 400 mg/kg body weight bitter melon (BM) extract suspended in mucin–water vehicle, or with vehicle (Control). Animals were segregated into four treatment groups, 10 animals in each group, according to strain (Lean or ZO) and treatment (Control or BM). Following six-week treatment periods, peripheral blood was collected from selected animals, followed by sacrifice, thoracotomy and mounting of isolated working heart setup. *Results*: Body mass of both Lean and ZO rats was unaffected by treatment, likewise, peripheral blood fasting glucose levels showed no significant treatment-related effects. However, some BM treatment-related improvement was noted in postischemic cardiac functions when Lean, BM-treated animals were compared to vehicle treated Lean control rats. Treatment of Lean, but not ZO, rats significantly reduced the magnitude of infarcted zone in isolated hearts subjected to 30 min of ischemia followed by 2 h of working mode reperfusion. Immunohistochemical demonstration of caspase-3 expression by isolated heart tissues subjected to 30 min of ischemia followed by 2 h of reperfusion, revealed significant correlation between BM treatment and reduced expression of this enzyme in hearts obtained from both Lean and ZO animals. The hierarchy and order of caspase-3 expression from highest to lowest was as follows: ZO rats receiving vehicle > ZO rats receiving BM extract > Lean rats treated receiving vehicle > Lean rats administered BM extract. Outcomes of analyses of peripheral blood content of cardiac-related analytics: with particular relevance to clinical application was a significant elevation in blood of ZO and ZO BM-treated, versus Lean rats of total cholesterol (high density lipoprotein HDL-c + low density lipoprotein LDL-c), with an inferred increase in HDL-c/LDL-c ratio—an outcome associated with decreased risk of atherosclerotic disease. *Conclusions*: BM extract failed to positively affect T2DM- and cardiovascular-related outcomes at a level suggesting use as a standalone treatment. Nevertheless, the encouraging effects of BM in enhancement of cardiac function, suppression of post-ischemic/reperfused infarct size extent and capacity to modulate serum cholesterol, will likely make it useful as an adjuvant therapy for the management of T2DM and related cardiovascular diseases.

## 1. Introduction

### 1.1. Type 2 Diabetes: Contributors to Pathogenesis

Type 2 Diabetes Mellitus (T2DM) is an obesity-associated metabolic syndrome, which has emerged as a disease with one of the most rapidly increasing rates of diagnosis worldwide at the time of this writing, particularly among populations in affluent nations with sedentary lifestyles and high incidence of obesity [1]. Metabolic syndrome is characterized by disrupted regulation of carbohydrate and fatty acid metabolism, with main features that include insulin resistance, dyslipidemia and hypertension [2]. Its pathogenesis occurs principally as a result of excessive expression of inflammatory cytokines by macrophages and fat cells (adipocytes). In a process where high levels of a major inflammatory cytokine, tumor necrosis factor-alpha (TNF-α), disrupts insulin signaling in a wide range of cells diminish the uptake efficiency of a particular cell for insulin and suppress the production of heat shock proteins (HSP), which are key regulators of primary immune responses [3]. Reduced HSP expression by a cell stimulates accumulation of lipid deposits and toxic protein aggregates, accumulation of which further exacerbates overexpression of pro-inflammatory mediators and geroconversion into senescent cell types. All of these factors can promote systemic inflammatory tissue damage [4,5]. Obesity and its associated morbidities are primary risk factors for T2DM and cardiovascular diseases. Epidemiological studies have demonstrated that heart failure in diabetic patients, depending on the gender, is 2–5 times more common than in non-diabetics [6]. Moreover, the occurrence of heart failure was found to be six times more frequent than in normotensive subjects with normal glucose utilization [7]. In the past decades, novel single drugs have been developed to combat T2DM. However, plant mixtures derived from traditional remedies, particularly Chinese medicine and Ayurveda, have emerged as increasingly favored clinical approaches to prevention and management of both diabetes and cardiovascular disorders.

### 1.2. Bitter Melon: Ethnobotany and Component of Therapeutic Phytochemicals

The bitter melon (*Momordica charantiais autochthon*), also known as balsam-pearl, bitter ground, bitter squash and kerala, is endemic to parts of South America, Africa, and Asia. It is a popular food item in India and China, where its raw, bitter fruit, tender leaves and shoots are consumed as pickles, or vegetable. Its medical uses include treatment for diabetes, hypertension, inflammation, fever, viral and bacterial infections and a diverse range of other disorders [8,9]. More than 200 active ingredients including mono- and triterpenes, carotenoids, alkaloids, steroids, lipids, and proteins have been isolated from different parts of this plant [10]. A principal therapeutic component of BM called momordicin has a long history of use in traditional Asian medicine [11]. Another bioactive compound called momocharin (charantin) is a steroid saponin mediates insulin-like effects, which has been identified as a major contributor to antidiabetic activities of the fruit [9]. A particularly interesting property of *M. Charantia* polysaccharides, is their capacity to augment production of the cytoprotective heat shock protein heme oxygenase-1 (HO-1), at levels demonstrated to abate major symptoms of streptozotocin-induced diabetes and diabetic nephropathy in a rat model [12]. This outcome is particularly significant in the context of the demonstration by the authors of the present report that HO-1 inducers from seeds of sour cherry (*Prunus cerasus*) mediated potent HO-1-dependent antidiabetic and cardioprotective properties [4,13].

### 1.3. Antidiabetic Effects of BM Extracts

Investigations of therapeutic capacities of the plant have demonstrated that it can alter fat and carbohydrate metabolism in ways that mitigate T2DM symptoms [14]. Related studies show its juice reduces the visceral fat content of rats fed with high fat diets, and also significantly reduces the body weight [15]. Recently, consumption of BM extract was shown to favorably alter the blood lipid profiles of Zucker Obese (ZO) rats, while increasing systemic insulin levels, ultimately resulting in decreased serum glucose levels in treated animals [16]. Conversely, other investigators failed to detect significant effects on blood sugar levels in streptozotocin-induced diabetic rats [11]. Moreover, human trials of BM consumption yielded inconclusive outcomes with respect to antidiabetic properties of the plant [17,18]. Thus, before its use can be incorporated into mainstream clinical practice, it will be necessary to conduct definitive demonstration of its efficacy in ameliorating onset and progression of T2DM. The present investigation evaluates effects of the plant on selected aspects of fat and sugar metabolism in a ZO rat model. These studies were extended to assess its capacity to mitigate ischemia/reperfusion-induced damage in the presence or absence of metabolic disruption.

## 2. Results

### 2.1. Body Mass

**Figure 1:**
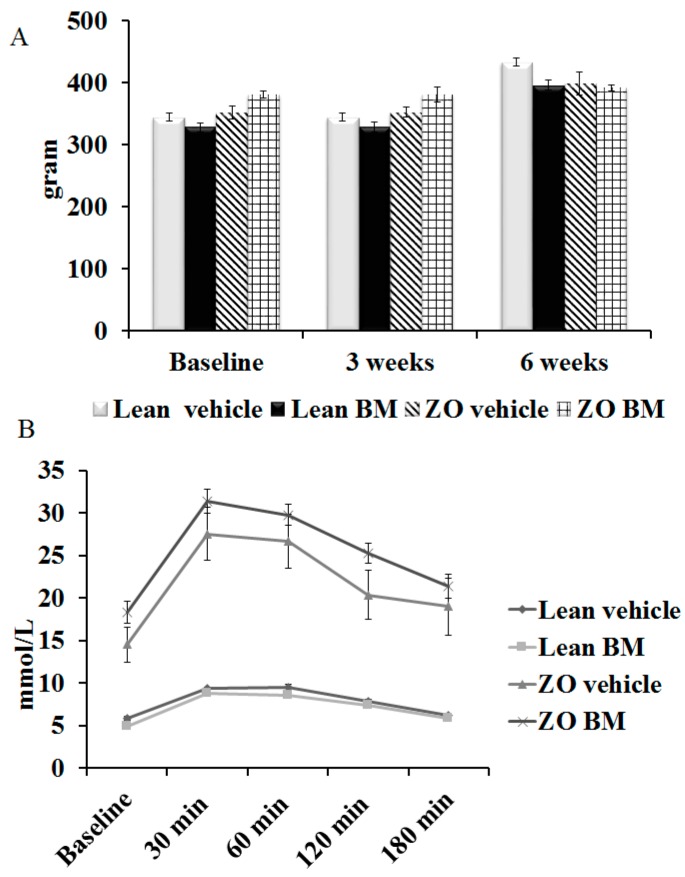
(**A**) Bitter melon (BM) extract effect on body weight. Six-week time course monitoring of body mass was conducted on Lean or Zucker Obese (ZO) rats (*n* = 10 animals in each group), fed daily by gavage, with mucin–water vehicle, or BM extract of 400 mg/kg body weight, at outset (Baseline) and the three-week and six-week time points. Results are provided as average body mass, in grams, ±SEM; (**B**) Effect of bitter melon extract on serum glucose metabolism. Lean or Zucker Obese (ZO) rats (*n* = 10 animals per group), fed daily by gavage, for six weeks with mucin–water vehicle, or BM extract of 400 mg/kg body weight, are evaluated for fasting serum glucose content immediately prior to oral administration of 3 g of glucose per kg of body weight (Baseline). Results are provided as average serum glucose content, in mmol/L of peripheral blood, ±SEM.

Time course evaluation of the effect of BM extract on body mass in treated animals is shown in Figure 1A. It demonstrates no significant changes in this variable as a correlate with treatment when baseline measurements are compared with the three-week and six-week time points, or between untreated versus treated animals at each time point.

### 2.2. Oral Glucose Tolerance Test

Outcomes of OGTT are shown in Figure 1B. As expected, serum glucose levels in both treated and untreated Zucker Obese rats were significantly elevated relative to levels observed in Lean animals at each time point; nevertheless, treatment with BM extract failed to exhibit correlation with significantly improved clearance of glucose in either obese or Lean animals.

### 2.3. Cardiac Function

**Figure 2:**
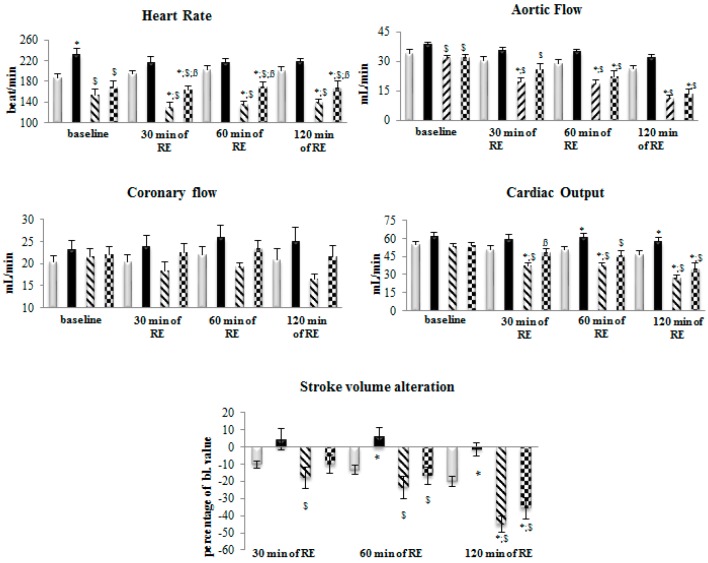
Effect of postischemic reperfusion on function of isolated working hearts from rats treated with bitter melon (BM) extract. Lean or Zucker Obese rats (*n* = 10 animals in each group), fed daily by gavage, for six weeks at 400 mg/kg body weight of BM, are sacrificed and thoracotomized, followed by mounting on isolated working heart setup. Hearts were subjected to 30 min of ischemia followed by 120 min of reperfusion. Cardiac functions including heart rate (HR), aortic flow (AF), coronary flow (CF), cardiac output (CO) and stroke volume alteration (devSV) were recorded before ischemia at time points of 0 (baseline), and 30, 60 and 120 min of reperfusion. Results are provided as average magnitude of each cardiac function value within a group of animals ± SEM. Significance level of difference between average outcome values shown in histograms, is *p* < 0.05, or less, indicated as follows: * *p* < 0.05 versus Lean control values; ^$^ *p* < 0.05 versus Lean BM values; ^ß^ *p* < 0.05 versus ZO values.

Figure 2 shows cardiac function results in hearts isolated from Lean and ZO rats, vehicle (control)-, or BM-treated, which are summarized as follows:

**Heart rate:** At baseline (time point 0), we have noticed slightly elevated heart rate values for hearts from BM-treated Lean rats; and slight bradycardia were seen in hearts obtained from ZO treated animals in the presence or absence of BM. After 30 and 60 min of reperfusion, a significantly lower heart rate for ZO animals, regardless of treatment, were seen compared to the heart rates observed from Lean animals. After 120 min of reperfusion, hearts originated from ZO (vehicle and BM treated) animals exhibited significantly lower heart rate in comparison with the Lean groups. However, hearts originated from ZO-BM group exhibited significantly higher heart rate compared to hearts from ZO vehicle treated group (*p* < 0.05).

**Aortic flow:** At baseline, small, but significantly lower aortic flow (AF) values were observed in hearts from both vehicle- and BM-treated ZO rats, relative to BM-treated Lean animals (*p* < 0.05). At 30, 60 and 120 min of reperfusion, AF values for hearts from both vehicle- and BM-treated ZO animals was significantly reduced relative to BM-treated Lean group (*p* < 0.05). At 30 min of reperfusion, AF for vehicle-treated ZO, but not BM-treated ZO animals was reduced relative to vehicle-treated Lean (*p* < 0.05), and at 60 and 120 min reperfusion time points. AF for vehicle- and BM-treated ZO animals were reduced relative to vehicle- and BM-treated Lean groups (*p* < 0.05). It has to be also noted that in both BM-treated groups a slightly higher AF value were detected in comparison with the vehicle treated group.

**Coronary flow:** No significant differences in coronary flow (CF) were observed between animal strains and between the BM and vehicle treated groups trough the experiments. However, during reperfusion the CF values of ZO hearts tend to be lower compared to the values of Lean animals and values of BM treated groups tend to be higher compared to vehicle treated groups.

**Cardiac output:** Comparison of cardiac output (CO) values between animal strains and treatment groups revealed no significant differences at baseline. Conversely, at the 30, 60 and 120 min time points, CO in hearts from vehicle-treated ZO rats were significantly reduced relative to both vehicle- and BM-treated Lean animals (*p* < 0.05). Moreover, at the 60 and 120 min reperfusion time points, CO measured in hearts from BM-treated Lean rats significantly exceeded CO in hearts from vehicle-treated Lean animals (*p* < 0.05). Finally, it was observed that at the 60 min of reperfusion, CO in hearts from BM-treated ZO rats, significantly exceeded values of this outcome variable measured in hearts from vehicle-treated ZO animals (*p* < 0.05), indicating cardioprotective properties of BM.

**Stroke volume alteration measured as percentage of value measured before initiation of reperfusion (baseline):** Significant decreases in this parameter were observed in hearts from vehicle-treated ZO rats at 30, 60 and 120 min of reperfusion relative to hearts from BM-treated Lean animals (*p* < 0.05), with a response pattern that suggested dose-responsive reduction in stroke volume with increasing reperfusion time. Moreover, at the 60 and 120 min of reperfusion, hearts from BM-treated ZO rats also exhibited significant shortening of stroke volume relative to hearts from BM-treated Lean rats (*p* < 0.05). Conversely, relative to vehicle-treated Lean rats, a small but significantly lower decrement in stroke volume shortening was observed in BM-treated Lean rats at the 120 min of reperfusion. Treatment of Lean animals with BM appeared to confer resistance to shortening of stroke volume throughout this experiment.

### 2.4. Infarcted Zone Extent

**Figure 3:**
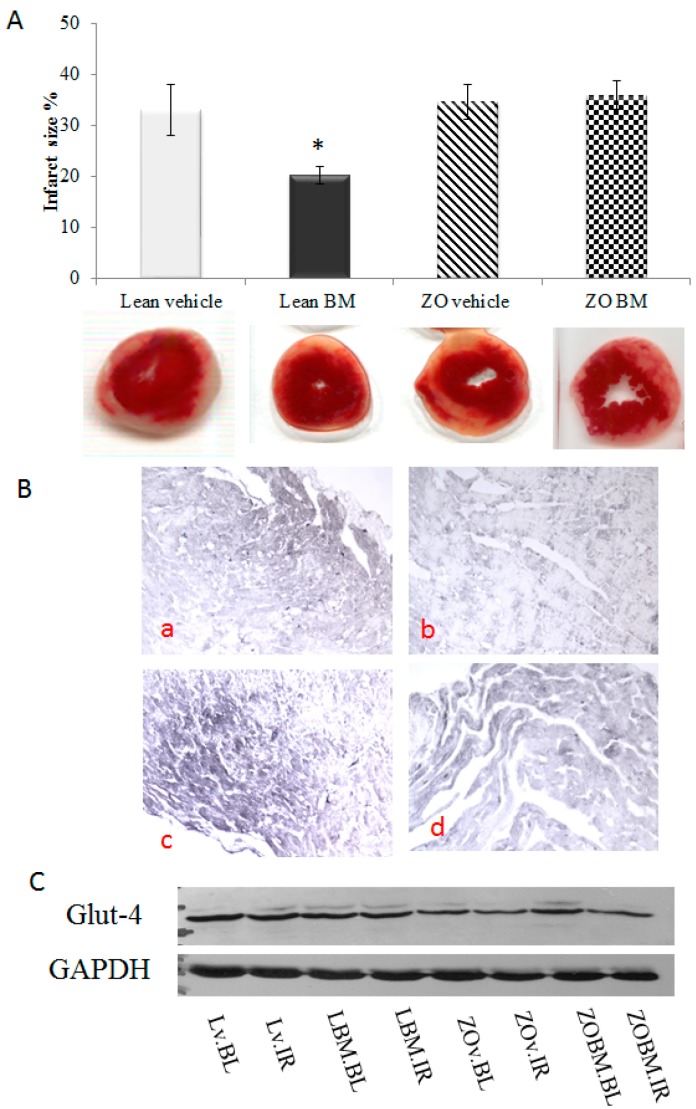
(**A**) Effect of bitter melon (BM) extract on postischemic cardiac infarct magnitude. Lean or Zucker Obese (ZO) rats (*n* = 4 animals in each group), fed daily by gavage, for six weeks with mucin–water (vehicle), or BM extract of 400 mg/kg body weight, are sacrificed and thoracotomized, followed by mounting on isolated working heart apparatus. Hearts were subjected to 30 min of ischemia and 2 h of reperfusion, followed by perfusion with 35 mL of 1% TTC solution via aortic cannula and stored for 24 h at −20 °C to allow cardiac tissue solidification. Next, 2–3 mm-thick sections of stained hearts were scanned, weighed planimetrically analyzed and infarct size were calculated. Outcomes were expressed as the ratio of the total infarcted tissue volume to volume of at-risk tissue ±SEM. Significance level of difference (*p*) between average outcome values shown in histograms, is *p* < 0.05, or less, indicated as follows: * *p* < 0.05 versus Lean IR values; (**B**) Effect of BM extract on postischemic expression of caspase-3 in isolated, ischemia/reperfusion (I/R)-injured rat hearts. Lean or Zucker Obese (ZO) rats (*n* = 4 animals in each group), fed daily by gavage, for six weeks with mucin–water vehicle, or BM extract of 400 mg/kg body weight, are sacrificed and thoracotomized, followed by mounting on isolated working hearts in a Langendorff apparatus. Hearts were subjected to 30 min of ischemia and 2 h of reperfusion, followed by formalin fixation and paraffinization. Then, 4.5 micron thick sections were subjected to immunohistochemical analyses for caspase-3. Images shown include: Lean rats treated with vehicle (**Ba**); Lean rats administered BM extract (**Bb**); ZO rats receiving vehicle (**Bc**); and ZO rats receiving BM extract (**Bd**); (**C**) Effect of Bitter melon (BM) extract on Glut-4 expression: Protein samples from hearts originated from Lean and ZO animals in the presence or absence of BM treatment were separated and probed with Glut-4 antibody. As it is depicted, the level of Glut-4 is lower in samples from ZO animals regardless of treatment. However, the BM treatment failed to alter the cardiac Glut-4 level.

The ability of BM extract to inhibit I/R injury-associated infarction of isolated rat hearts is shown by analysis of TTC-stained sections of cardiac tissue in Figure 3A. These data demonstrate that infarct zone magnitude in hearts taken from BM-treated Lean rats were significantly lower than the infarct zone extent exhibited by vehicle treated Lean rats, confirming the cardioprotective role of BM (*p* < 0.05). Conversely, the extract failed to mediate significant suppression of I/R-associated infarction magnitude manifested by hearts isolated from ZO rats.

### 2.5. Immunohistochemical Demonstration of Caspase-3 Expression in the Cardiac Tissue

Photomicrographs of four representative immunohistochemical sections are shown in Figure 3B. Tissue expression of caspase-3 is indicated by darker-staining areas. IHC results are presented to provide a qualitative overview only, of correlation between treatment received by test animals and expression of this enzyme in heart tissue. The highest expression was observed in ZO rats receiving vehicle (3Bc), followed by ZO rats receiving BM extract (3Bd), and followed by Lean rats treated with vehicle (3Ba), with lowest caspase-3 expression in tissue from Lean rats administered BM extract (3Bb).

### 2.6. Glut-4 Expression

A representative Western blot is shown in Figure 3C. Thus, a decreased Glut-4 expression was found in hearts originated from ZO animals compared to Lean hearts. Interestingly, BM failed to influence the level of Glut-4 in either of the treated groups in comparison with vehicle treated controls.

### 2.7. Peripheral Blood Content of Cardiac-related Analytes

**Figure 4:**
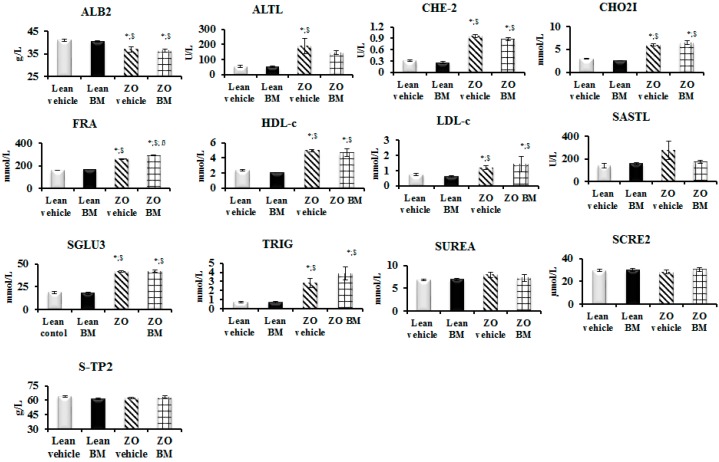
Effect of bitter melon (BM) extract on serum cardiovascular analytes in peripheral blood. Lean or Zucker Obese (ZO) rats (*n* = 10 animals in each group), fed daily by gavage, for six weeks with mucin–water vehicle, or BM extract of 400 mg/kg body weight. Peripheral blood samples collected from the jugular vein at the end of treatment periods, was analyzed for albumin (ALB2), alanine aminotransferase (ALTL), cholinesterase (CHE-2), total cholesterol (CHO2I), fructosamine (FRA), high-density lipoprotein cholesterol (HDL-c), low-density lipoprotein cholesterol (LDL-c), aspartate aminotransferase (SASTL), serum glucose (SGLU3), triglycerides (TRIG), serum urea (SUREA), serum creatinine (SCRE2) and total protein (S-TP2). Outcomes are expressed as the average content of each analyze in mmol/L ± SEM. Significance level of difference (*p*) between average outcome values shown in histograms, is *p* < 0.05, or less, indicated as follows: * *p* < 0.05 versus Lean IR values; ^$^ *p* < 0.05 versus Lean BM values; ^ß^ *p* < 0.05 versus ZO values.

Results of the analysis of peripheral blood collected from rats following six weeks of gavage treatment with BM extract or vehicle are shown in Figure 4. Relative to Lean rats receiving vehicle, the average level of albumin in blood of ZO animals was significantly lower (*p* < 0.05). Moreover, relative to Lean control animals blood content of alanine aminotransferase was significantly elevated in vehicle treated ZO animals (*p* < 0.05), but not extract-treated (ZO BM) subjects (*p* < 0.05). Additionally, blood of ZO and ZO BM animals exhibited significantly elevated levels of total cholesterol (CHO2I), fructosamine (FRA), low-density lipoprotein cholesterol (LDL-c), triglycerides (TRIG), cholinesterase (CHE-2), high-density lipoprotein cholesterol (HDL-c) and serum glucose (SGLU3), compared to Lean control animals (*p* < 0.05).

## 3. Discussion

### 3.1. Major Medical Challenges and Modern Countermeasures

The extent to which obesity-related diseases have adversely impacted health of the world’s population, particularly in societies with lifestyles that have become progressively sedentary, reveals effects of the great plagues of past centuries. Cardiovascular syndromes and their comorbidities, particularly type 2 diabetes figure prominently among these medical problems. Interventions, capable of providing cost-effective means to prevent and manage such metabolic derangements, have thus emerged as a major priority for basic and clinical research. Approaches to the development of these techniques have become increasingly sophisticated and may be broadly divided into two major strategies. One approach classed generically as “orthodox” rely heavily on single molecule compounds created to affect cellular processes at critical checkpoints in signaling cascades that have become pathologically altered in ways that adversely impact normal tissue and organ function. Examples include corticosteroids such as prednisone which affect gene expression to counteract inflammatory tissue damage associated with severe chronic illness, but disrupt normal homeostatic activity so as to themselves induce chronic disease [19]. Other examples include the cyclooxygenase-2 (COX-2)-specific non-specific anti-inflammatory drug rofecoxib (Vioxx), which proved to be enormously effective in providing powerful but transitory relief of pain in rheumatoid arthritis, but disrupted prostaglandin biosynthesis at levels that resulted in severe and occasionally fatal cardiotoxicity [20].

### 3.2. Rediscovery and Advanced Use of Traditional Medical Practices

In recent years, a rediscovery of ethnobotanical methods for preventing and treating disease, has emerged as an increasingly potent parallel form of medical practice to prevailing orthodoxy, which is often reliant on drugs that are toxic, costly and ultimately only palliative in long-term effect. Interest in traditional medical practices has been strongly stimulated by demonstration that their use avoids many of the problems associated with orthodox healthcare practices and in many cases exhibit high therapeutic potency, with minimal occurrence of adverse side effects. These trends have been considerably boosted by current emphasis from the U.S. government on developing medical strategies that attack disease at its roots, rather than merely providing analgesic and palliative relief from symptoms. These encouraging trends are reflected by the U.S. NIH’s current policy directive (The Precision Medicine Initiative^®^ (PMI): A White House policy directive, 20 January 2015). This policy initiative has stimulated government support for “Complementary and Integrative Medicine” (CAIM) programs, which are highly innovative uses of modern biotechnology to characterize and improve clinical applications of Ayurveda, Traditional Chinese and a wide range of other ancient medical methods that have historically proven capacities for alleviating disease. These traditions are often termed “holistic” based on their capacity to align dimensions of both physical and spiritual experiences of the human condition. Nevertheless, in order to optimize safety and efficacy of CAIM-based interventions, it is necessary to rigorously evaluate the specific physiological effects of such agents on disease pathophysiology. The present investigation, was undertaken to provide researchers and clinicians with a perspective on how bitter melon, a plant used for centuries as a traditional treatment for diabetic conditions, affects aspects of glucose metabolism that become deregulated as underlying elements of type 2 diabetes [21].

Bitter melon extract has recently been demonstrated to exert potent cardioprotection through the ability of its polysaccharide components to modulate the activity of the inflammation-associated transcription factor, nuclear factor kappa B (NF-κB) in ways that enhance cellular antioxidant defenses [22]. These studies demonstrated that the pretreatment of rats with these compounds prior to isoproterenol-induced myocardial infarction, inhibited inflammatory cytokine production in cardiovascular tissue and augmented production and biological effect of non-protein sulfhydryls, catalase, superoxide dismutase and Bcl-2 (an anti-apoptotic protein), while decreasing expression of the apoptotic markers caspase-3 and BAX, along with inducible nitric oxide synthase, nitric oxide and myeloperoxidase [22]. The major effects of alteration by components of the extract, of the above-mentioned compounds and the cellular physiologic processes in which they participate, included significantly lower lipid peroxidation and overall oxidative stress levels, with resulting outcomes such as reduced extent of cardiac infracted zones, inhibition of infarction-associated increases in heart-weight-to-body-weight ratio.

### 3.3. Effects of Bitter Melon Extract on Cardiovascular and Metabolic Parameters in a Rat Model

The data in Figure 1A that show the results of time-course evaluation of the effect of BM on body mass do not support the use of the extract as a weight control measure within the timeframe the measurements were conducted, since treatments failed to correlate with significant changes in weight of the animals. This conclusion is subject to a caveat that such changes may be observed with longer treatment periods, and/or in combination with other antidiabetic agents. As expected, serum glucose levels measured in blood of Lean rats were significantly lower than those in ZO animals at each time point measured (Figure 1B). Nevertheless, the absence of significant BM-associated improvement in glucose utilization in either the Lean or ZO animals suggests that the extract may prove inefficient in aspects of diabetes treatment directly related to utilization of glucose. The effects of BM extract and rat strain on cardiac function are shown in Figure 2. Contributors to heart rate are multifactorial, the apparent stimulatory effect of BM and elevated rates in Lean versus ZO animals cannot be comprehensively explained by data presented here. This effect, nevertheless, may be indicative of a cardioprotective influence of the extract, since exenatide, an incretin mimetic drug, used to improve normal glucose metabolic function in T2DM patients through its agonist effects on the glucagon-like peptide-1 receptor, is observed to elevate heart rate in human T2DM patients [23]. As expected, treatment of Lean rats with BM extract correlated with improved aortic flow relative to ZO/untreated animals. This suggests that the agent may augment the amelioration of vasoconstrictive influences, an effect that the small, but significant change in stroke volume alteration by Lean BM, versus ZO animals. Several phytochemicals present in BM exhibit potent antioxidant effects, which are likely contributors to improved aortic flow and alteration of cardiac stroke volume shown in Figure 2 [24]. Recent studies by other investigators support this possibility [25] and form the basis for ongoing characterization of the plant’s bioactive properties by authors of the present report. The capacity of bioactive components of BM to mitigate ischemia/reperfusion-associated damage to cardiac tissue has been previously demonstrated in studies revealing that polysaccharides produced by the plant, inhibit cerebral ischemia/reperfusion injury [26,27]. Within the present study, evaluation of the plant’s ability to mitigate ischemia-reperfusion injury was conducted in experiments demonstrating bitter melon extract effects on ischemia/reperfusion-associated infarct size magnitude. Outcomes shown in Figure 3A, provide a particularly striking demonstration of a cardioprotective effect of the extract. This is revealed by approximately 1/3 lower extent of infarcted area in left ventricular tissue taken from BM-treated animals, versus hearts from rats receiving mucin–water as vehicle treated control. Conversely, and unexpectedly, a similar effect was not observed in hearts taken from ZO rats. This result may be due to the defective leptin receptor, which is the genetic feature that defines the ZO strain. Specifically, one or more compounds in BM may act through this receptor in ways that inhibit ischemia or diabetes-related processes that promote infarction of cardiac tissue. This possibility is nevertheless speculative and cannot be definitively determined based on data in the present study. Outcomes of immunohistochemical analyses for caspase-3 expression shown in Figure 3B, which may be produced at pathological levels in cardiac tissue of a diabetic rodent model [28] and contributes to muscle atrophy in human T2DM patients [29]. The IHC results are therefore consistent with effects on cardiac caspase-3 production in obesity-related diabetes and a possible therapeutic effect of bitter BM extract. Specifically, higher expression of the enzyme is observed in tissue from ZO rats, versus Lean, and treatment with the extract correlated with reduced caspase-3 production for both strains of rat. Earlier, we have demonstrated that the ability of phytochemicals to restore the Glut-4 level in cardiac tissue may contribute to their cardioprotective properties. Consistently, a lower level of Glut-4 was observed in ZO animals accompanied by poorer cardiac recovery. However, BM treatment failed to enhance the level of this protein indicating that the cardioprotective effect seen in BM treated Lean animals is being mediated via different mechanisms.

Outcomes of analyses for cardiovascular function-related serum analytes (Figure 4), with particularly promising relevance to clinical use of bitter melon, are effects of the extract on peripheral blood content of HDL and LDL cholesterol. The plant contains lauric acid a dietary triglyceride, which is a potent regulator of blood cholesterol levels, both as HDL-c and LDL-c [30], with greater augmentative effect on the “good” cholesterol (HDL-c) than any other dietary fatty acid evaluated at the time of this writing [31]. Moreover, other compounds in the plant inhibit CYP4, an enzyme that increases hydroxylation of lauric acid in liver, kidney, and brain of diabetic rats [32]. Thus, extract of the melon is a lauric acid source and also inhibits physiological degradation of the compound derived from diet which is increased in diabetes, as described by Raza and colleagues [33]. Thus, as shown in Figure 4, BM extract elevates the HDL/LDL ratio, an effect observed to correlate with reduced atherosclerotic risk [34].

## 4. Materials and Methods

### 4.1. Animals

Animals used in the present study were male Lean and Zucker Obese (ZO) rats with an average weight of 200 ± 15 g and 230 ± 10 g, respectively. They were provided with standard rodent chow pellets (Formulab Diet 5008, LabDiet, St. Louis, MO, USA) and water ad libitum with free access to water and housed at an ambient temperature of 25 ± 2 °C, with a relative humidity of 55% ± 5%, and a 12-h light-dark cycle. All animals were acclimatized for one week prior to initiation of experiments and treated according to the “Principles of Laboratory Animal Care” formulated by the National Society for Medical Research, and the “Guide for the Care and Use of Laboratory Animals” prepared by the National Academy of Sciences and published by the National Institutes of Health (NIH Publication no. 86–23, revised in 1996). Treatment of the animals and experimental design was approved by the Institutional Animal Care and Use Committee of the University of Debrecen (UD), Debrecen, Hungary. UD ethical committee approval certificate number: 3/2012/DE MÁB.

### 4.2. Treatment Protocol

Bitter melon extract for the treatments was made by suspending the content of commercially available bitter melon capsules (manufactured by ISI Brands Inc., American Fork, UT, USA). Suspending medium (vehicle) was a commixture of 2% hydroxyethylcellulose solution (mucin) and water (1:4). Rats were randomly segregated into four different groups as described below: GROUP I: Lean rats, gavage-treated with mucin–water vehicle. GROUP II: Lean rats, gavage-treated with 400 mg/kg body weight of BM extract. GROUP III: Zucker Obese rats, gavage-treated with mucin–water vehicle. GROUP IV: Zucker Obese rats, gavage-treated with selected doses of BM extract. All animals were treated for a time period of six weeks. Body mass was measured before, during and at the end of the treatments. At the 4th week of the treatment, oral glucose tolerance assays were performed.

### 4.3. Oral Glucose Tolerance Test

Feeding of each animal was discontinued 12 h before evaluations. After this fasting period, 3 g glucose per kg of body weight dissolved in water was orally administered to the rats. Blood sugar levels were assessed using peripheral blood collected from tail veins before glucose administration and 30, 60, 180 min following. Blood concentration of glucose was assessed by Accu Chek Active glucose monitoring device (Roche Diabetes Care GmbH, Mannheim, Germany).

### 4.4. Isolated Working Heart Preparation and Cardiac Function Assessments

Following the six-week of treatment period, rats were anesthetized with an intraperitoneal pentobarbital sodium injection (60 mg/kg), with heparin as an anticoagulant (1000 U/kg). After induction of deep anesthesia, chest cavities were opened, hearts were excised and placed in ice cold modified Krebs-Henseleit bicarbonate (KHB) buffer (118 mM NaCl, 5.8 mM KCl, 1.8 mM CaCl_2_, 25 mM NaHCO_3_, 0.36 mM KH_2_PO_4_, 1.2 mM MgSO_4_, and 5.0 mM Glucose) to prevent damage of cardiac tissue. After excision, aortas were cannulated and each heart was perfused with modified Krebs-Henseleit buffer at a filling pressure of 100 cm of water (10 kPa), using the non-working mode of a Langendorff apparatus for 5 min to flush out blood from the heart. During the washout period, pulmonary veins were cannulated, and heart functions were assessed in working mode at a filling pressure of 17 cm (10 kPa) of water with KHB buffer. A total of 10 min of working mode activity was sustained to stabilize the cardiac activity. At the end of 10 min of working mode perfusion, baseline cardiac parameters including heart rate (HR), aortic flow (AF), coronary flow (CF), cardiac output (CO) and stroke volume alteration (devSV) were registered. Next, 30 min of ischemia was induced by closing the atrial inflow and aortic outflow lines. At the end of ischemic periods, reperfusion was initiated by opening the atrial inflow and aortic outflow cannulas. The first 10 min of reperfusion was conducted in Langendorff mode to prevent development of fatal ventricular arrhythmias. If ventricular fibrillation was observed at the onset of reperfusion, the heart was defibrillated with a square wave impulse. After the first 10 min of Langendorff reperfusion, hearts were switched to working mode for 110 minutes. At 30, 60 and 120 min of the repefusion period, cardiac parameters were recorded to monitor the postischemic recovery of the myocardium. A continuous pressure signal was recorded during the whole experiment with the help of a pressure transducer (ADInstruments, PowerLab, Castle Hill, Australia). HR and AOP were calculated from the continuously recorded pressure signal. AF was measured by a calibrated flow meter, while CF was assessed by time-collecting the coronary effluent. Cardiac output (CO) was calculated as the sum of AF and CF, while stroke volume (SV) was the ratio of CO and HR. Stroke volume alteration was calculated as a ratio of SV and baseline SV [24].

### 4.5. Infarct Size Measurement

To monitor the degree of infarction, triphenyl tetrazolium chloride (TTC) (Sigma-Aldrich, Inc., St. Louis, MO, USA) staining was carried out. At the end of the 2 h of reperfusion, hearts were perfused with 35 mL of 1% TTC solution via the aortic cannula. After 10 min, hearts were placed at −20 °C for 24 h, to allow each heart to solidify. A total of 2–3 mm-thick sections were made from the stained frozen hearts. Sections were subsequently scanned on an Epson J232D flat-bed scanner (Seiko Epson Corporation, Nagano Japan), blotted dry and weighted. The infarcted area (identifiable by white coloration) and the risk area (entire scanned section) were measured using planimetry software (version 1.46, Image J, National Institutes of Health, Bethesda, MD, USA). Estimates of infarcted zone magnitude were subsequently obtained by multiplying infarcted areas by weight of each section. The resulting numbers represent weight of the risk zone and the infarcted zone. Infarct size was estimated as a ratio of the weight of infarcted tissue and the weight of risk zone (whole heart). The entire area of each section was considered to be an infarction risk zone, while the numerical extent of each infarcted area (staining white by TTC), was planimetrically calculated and multiplied by the weight of the section. Outcomes were expressed as the ratio of the total infarcted tissue volume to volume of at-risk tissue [24].

### 4.6. Immunohistochemistry

At the end of the ischemia and reperfusion (I/R) experiments heart tissues were fixed in 4% formalin for 24 h at 4 °C, embedded in paraffin, and cut into 4.5 micron thick sections. All tissue sections were deparaffined in xylene and rehydrated in a graded ethanol series. Antigen retrieval was accomplished by boiling the slides in 100 mM sodium citrate (pH 6.0) for 2 min in a pressure cooker, cooled with tap water, and then placed in phosphate buffer saline (PBS). Endogen peroxidases were blocked with 3% H_2_O_2_ containing PBS for 20 min at room temperature. To block aspecific binding, 1% BSA in PBS (pH 7.4) was used for 20 min at room temperature. Following the blocking, the slides were washed with PBS and incubated overnight with a caspase-3 primary antibody (#9662 Cell Signaling Technology, Boston, MA, USA) diluted in PBS (1/600) at 4 °C. The slides were washed in PBS twice and incubated with the horseradish peroxidase-conjugated secondary antibody (Super Sensitive One-Step Polymer-HRP Detection System, Bio Genex, Fremont, CA, USA) for 40 min at room temperature. After washing with PBS and sodium acetate (pH 6.6 and pH 6.0), the slides were incubated with DAB solution (Sigma Aldrich, Schnelldorf, Germany) to visualize the antibody binding. A positive reaction was indicated by development of a brown stain. The reaction is stopped by placement in a coplin jar filled with tap water. After washing, the slides were incubated with Tris-CoCl_2_ solution (pH 7.4) for increase the contrast between the stained and unstained parts of the tissue. The slides were washed with Tris-buffer saline (TBS pH 7.4), air-dried, and subsequently covered with mounting medium and glass slide covers. Moviol solution was used as mounting medium. Images were captured by a Zeiss Axioskop microscope (Carl Zeiss Microscopy GmbH, München, Germany) [24].

### 4.7. Blood Chemistry

Following six-week treatment with BM extract or vehicle and prior to sacrifice, peripheral blood was collected from left external jugular vein of each animal. Analysis for selected serum analytes was conducted using the Cobas 8000 modular analyzer series (Roche Diagnostics GmbH, Mannheim, Germany). The samples were assayed for content of albumin (ALB2), alanine aminotransferase (ALTL), cholinesterase (CHE-2), total cholesterol (CHO2I), fructosamine (FRA), high-density lipoprotein cholesterol (HDL-c), low-density lipoprotein cholesterol (LDL-c), aspartate aminotransferase (SASTL), serum glucose (SGLU3), triglycerides (TRIG), serum urea (SUREA), serum creatinine (SCRE2) and total protein (S-TP2). All testing was conducted in the Department of Laboratory Medicine, University of Debrecen, Debrecen, Hungary.

### 4.8. Western Blots

Approximately 300 mg of heart tissue was lysed in 1 mL isolating buffer (25 mM Tris-HCl, 25 mM NaCl, 1 mM orthovanadate, 10 mM NaF, 10 mM pyrophosphate, 10 mM okadaic acid, 0.5 mM EDTA, 1 mM PMSF, and 1x protease inhibitor cocktail) using a polytron homogenizer. Homogenates were centrifuged at 2000 rpm at 4 °C for 10 min. Supernatant was transferred to a new tube and further centrifuged at 10,000 rpm at 4 °C for 20 min; the supernatant was used as cytosolic extract. The protein concentration was determined by a BCA Protein Assay Kit (Thermo Scientific, Rockford, IL, USA) using bovine serum albumin (BSA) as the standard. Samples were mixed with Laemmli buffer and boiled for 10 min. A total of 100 μg of protein from each sample were loaded and separated on 12% SDS-PAGE gel (Sigma Aldrich, Schnelldorf, Germany) and then transferred to polyvinylidene difluoride (PVDF) membranes (Bio-Rad Laboratories, Hercules, CA, USA). After blocking the membranes with 5% of nonfat dry milk in Tris-buffered saline with 0.1% Tween 20 (TBST) for 1 h, membranes were incubated with primary antibody solution at 4 °C overnight (Glut-4 1/500, Abcam, Cambridge, UK; GAPDH (1/20,000, Cell Signaling Technology, Boston, MA, USA). Membranes were washed with TBST 3 times and incubated with horseradish peroxidase (HRP)-conjugated secondary antibody solution (1/2000, Cell Signaling Technology, Boston, MA, USA) for 1.5 h at room temperature. After washing, the membranes were developed using Luminate Forte Western HRP substrate (Millipore, Billerica, MA, USA) and the chemiluminescence was detected on X-ray films (Agfa, Mortsel, Belgium). After scanning the films, the band intensities were measured with Image J.

### 4.9. Statistical Analyses

All data are presented as the average magnitudes of each outcome in a group ± standard error of the mean (SEM). Statistical analysis was performed using one-way analysis of variance (ANOVA), followed by Kruskal–Wallis multiple comparison tests with GraphPad Prism software for Windows (GraphPad Software Inc., La Jolla, CA, USA). Probability values (*p*) less than 0.05 were considered statistically significant.

## 5. Conclusions

The present study, which evaluated outcome measures relevant to obesity-related metabolic syndrome and related cardiovascular effects, revealed expected correlations between animal strain and each effect measured. Specifically, Lean rats exhibited healthy physiologic status, which was generally augmented by administration of bitter melon extract and, for most variables, was significantly improved in comparison to Zucker Obese animals. Somewhat disappointingly, no significant correlation was noted between treatment with the extract and favorable alterations in body mass, serum glucose and in infarcted area extent in ZO rats. Nevertheless, extract intake-related effects on cardiac function related to vascular tone and improved blood flow, along with significant reduction of infracted zone magnitude in Lean animals, reduced caspase-3 expression in cardiac muscle and improved peripheral blood content of HDL-c are encouraging. These results suggest that although BM extract is probably unsuitable as a stand-alone therapy, use of this plant has potential to significantly enhance the effectiveness of drugs currently prescribed for obesity-related disorders, including type 2 diabetes and related cardiovascular diseases. Use of the plant as adjuvant therapy is expected to be particularly useful for minimizing extent of infarcted heart tissue and improving blood circulation following ischemia-reperfusion injury to the organ. The above conclusions are subject to the caveat that with longer treatment periods and/or different dose regimens, clinically relevant improvements in the evaluated outcome measures may be obtained. Investigations by authors of this report, which extend the results shown here, are ongoing.
